# Cross-Sectional Study on the Comparative Assessment of Mandibular Anesthesia (Inferior Alveolar Nerve Blockage) Manual Skills Shaping among Dentists on Plastic and Biomaterial Models

**DOI:** 10.3390/dj10070124

**Published:** 2022-07-04

**Authors:** Yuriy Vasil’ev, Ekaterina Diachkova, Hadi Darawsheh, Artem Kashtanov, Ekaterina Molotok, Beatrice Volel, Artem Batov, Olesya Kytko, Rinat Saleev, Gulshat Saleeva, Laysan Saleeva, Irina Smilyk, Natalya Tiunova

**Affiliations:** 1 N.V. Sklifosovsky Institute of Clinical Medicine, I.M. Sechenov First Moscow State Medical University (Sechenov University), 8-2 Trubetskaya St., 119991 Moscow, Russia; katenochek.m@mail.ru (E.M.); volel_b_a@staff.sechenov.ru (B.V.); batov3003t@gmail.com (A.B.); kytkodoc@yandex.ru (O.K.); 2 E.V. Borovsky Institute of Dentistry, I.M. Sechenov First Moscow State Medical University (Sechenov University), 11 Mozhaiskiy Val St., 121059 Moscow, Russia; 3 Institute of Anatomy “Skolkovo”, Skolkovo, 42-1 Bolshoy Boulevard St., 121205 Moscow, Russia; hadi.darawsheh@gmail.com (H.D.); kashtanov_artem@mail.ru (I.S.); 4 Dentistry Faculty, Kazan State Medical University, 49 Butlerova Street St., 420012 Kazan, Russia; rinat.saleev@gmail.com (R.S.); dr_ochnev001@mail.ru (G.S.); rin-gul@mail.ru (L.S.); 5 Dentistry Faculty, Privolzhsky Research Medical University, Minin and Pozharsky Sq., 603950 Nizhny Novgorod, Russia; natali5_@list.ru

**Keywords:** inferior alveolar nerve blockage, training dentist, plastic model, LIKERT scale, cadaver

## Abstract

Background: Providing regional anesthesia skills shaping remains relevant nowadays. A number of studies show that dentists have difficulties with these working independently. The study aim is the comparative analysis of the results of mandibular anesthesia (IANB) manual-skills shaping among dentists on plastic models and cadavers. Methods: In total, 999 participants were training in the skills of mandibular anesthesia from 2017 to 2021. The participants were divided in a random way into two groups: 700 participants were trained on plastic models, and 299 were trained on the cadaver material. After a lecture on the clinical and anatomical guidelines for IANB, a demonstration of the technique was provided, with subsequent testing of the injection technique. Satisfaction with the aspects of the training was assessed using the Likert scale. Results: the analysis of average values showed that participants from the group in which the manual skills were practiced on cadavers were more satisfied with the main aspects of the training, according to the sum of the main criteria of the modified scale. Conclusions: The important advantages of cadaver educational technology are that the sensations of tissue resistance are identical to natural ones, the individuality of each object, and the possibility of the visual study of the anesthesia technique, by dissection of the needle course and the location of the anesthetic depot.

## 1. Introduction

Local anesthesia, including mandibular anesthesia (inferior alveolar nerve block—IANB), is one of the stress factors for dentists, and a stressful situation not only for the patient but also for the dentist. This is due to the patient’s somatic pathology, and the doctor’s concerns about the development of undesirable side effects associated with a reaction to a local anesthetic [1,2,3,4]. In previously performed studies by Vasil’ev Yu. et al., 2020, and Rabinovich S.A. et al., 2017, it was mentioned that there are decreasing tachycardia and saturation as the cause of IANB failure [5,6]. One of the ways for reducing doctors’ stress and improving their manual-skills shaping is training programs in virtual reality (VR), which have been developed in recent years [7,8,9]. Liaw S.Y. et al., 2022, drew a conclusion that VR-tech learning could provide the similar performance outcomes as conventional simulation training [10]. The main difficulty in implementing VR-tech learning is the non-uniqueness of the results, due to the complexity of teaching VR technologies for manual-skills shaping. This fact led to a VR-tech training replacement, with phantoms of various levels of complexity, including interactive semi-anthropomorphic and cadaver ones [11,12]. Moreover, in accordance to Collaço E. et al., 2021, most doctors prefer to “feel in touch” what they do [13]. Thus, in research by Huri G. et al., 2021, the superiority of cadavers-based training on VR-tech in shoulder surgery was mentioned [14]. In a study by Stefanidis D. et al., 2013, it was mentioned that there were no differences in the perceived similarity to live-patient surgery in a study on pigs and human cadaver material [15]. Activities, including VR-tech, augmented reality (AR), 3D printing of models, and cadavers nowadays in the educational process, open new vistas in manual-skills shaping [16,17]. However, in the dentistry section there is lack of such studies—there are no results at PubMed when searching for “inferior alveolar nerve blockage AND stress AND dentist”, “inferior alveolar nerve blockage AND cadaver AND training”, and “inferior alveolar nerve blockage AND cadaver AND phantoms”.

The preclinical stage plays an important role in the education of a doctor of any specialty [18]. Thus, the study aim was to evaluate the results of regional-anesthesia manual-skills shaping on the mandible by dentists, based on a questionnaire using two training methods: cadavers and on dental simulators. The null hypothesis was ‘the IANB manual skill shaping on cadavers is associated with higher total training satisfaction than on plastic models.

## 2. Materials and Methods

The cross-sectional study was conducted to analyze the results of the development of manual-skills shaping of inferior alveolar nerve block by the 999 participants (dentists and students in the “Dentistry” specialty), from 2017 to 2021. The study was performed on the basis of the various dental training centers in Moscow, Kazan, and Nizhny Novgorod and the training center “Institute of Anatomy” (Skolkovo). There were no differences in training conditions in different centers. The study results were analyzed with the anonymous online questionnaire (after each training participants filled it).

For inclusion and exclusion criteria, initially all participants performed Lemyr-Tessier-Fillion PSM-65 test. This scale is used to measure phenomenological structure of experienced stress. The sum of all answers is calculated (‘integral indicator of mental tension’ (IIMT)). The higher the IIMT, the higher the level of psychological stress.

- IIMT more than 155 points means a high level of stress, indicates a condition maladaptation and mental discomfort, the need to use a wide a range of means and methods for reducing neuropsychic tension, psychological relief, and changes in the style of thinking and life.
- IIMT in the range of 155–100 points means an average level of stress.
- Low-stress level (IIMT less than 99 points) indicates a state of psychological adaptation to workloads.

The inclusion criteria for participation in the study:

- to have filled and signed informed consent;
- dental specialists (students of dentistry faculty or practicing dentists);
- to have Lemyr-Tessier-Fillion PSM-25 test score more than 155.

The exclusion criteria:

- the participants wish to exclude their data;
- to have Lemyr-Tessier-Fillion PSM-25 test score less than 155.

Variables:

- outcomes—marks for all 8 questions of Lickert scale from 1 to 5 mean;
- exposures—training course in IANB for dentists (two ways: cadavers and plastic models);
- potential confounders—the presence of previous successful experience in IANB in clinical practice (these participants were not included in final sample size of our research), gender, and age (participants were balanced according PSM-25, in this case these confounders had minimal effect);
- possible effect modifiers were not found.

Disposable dental syringes “Artiject” (LLC “RusPharm”, Moscow, Russia) with a 4% Articaine Inibsa (Inibsa Dental, Barcelona, Spain) were used in the work. This injection system is equipped with a needle protector to prevent accidental injuries to the student’s hands. Previously, each participant was trained to conduct an aspiration test: the syringe was fixed in the rubber protector of the cartridge to create negative pressure inside when the piston was pulled back. The visualization of the procedure was achieved by immersing the needle in a red liquid. When the colored solution got into the cartridge, the training was considered effective. It allows to focus on skill shaping and to exclude possible auto-injury with a needle.

**Figure 1:**
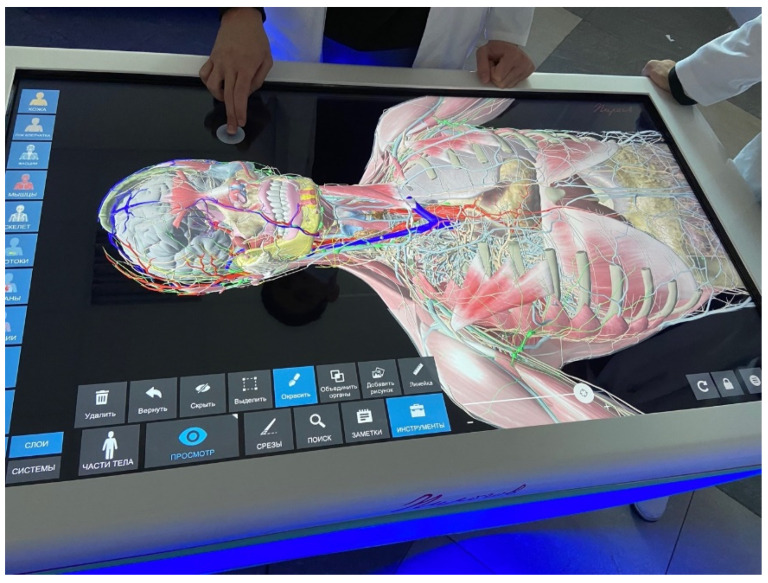
The theoretical part of training on an interactive anatomical table.

**Figure 2:**
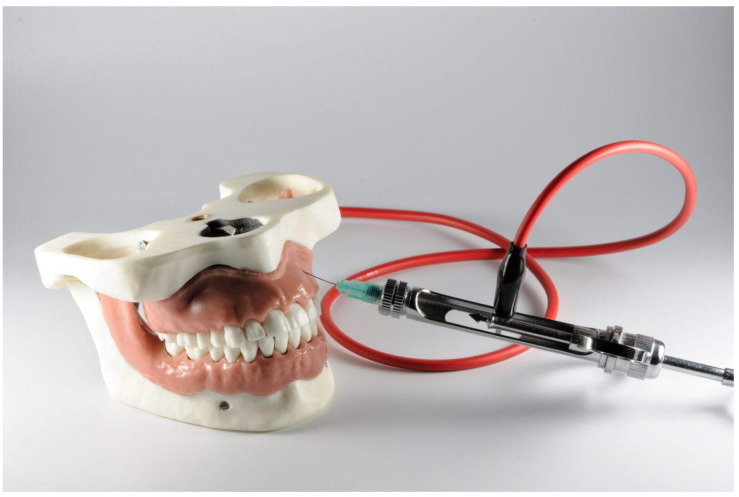
Dental simulator for the local anesthesia trainings, Energiyalab, Russia.

The trainings were carried out according to methodology:

1. Short lecture on clinical and anatomical guidelines for IANB: clinical anatomy of the maxillofacial area was presented at the interactive anatomical table «Pirogov» (LLC “Development”, Samara, Russia (Figure 1).
2. Local anesthesia landmarks were explained at the skull: these landmarks are usually used during IANB. In presented study, the following technique was used: the condyle process, the angle of the mandible and the anterior edge of the mandible branch are determined by palpation. A triangle is formed between these points, in the middle of which the middle finger of the hand is moved, the location of which is projected onto the mandibular foramen.
3. On a plastic model in the position of the open mouth, the participants palpate a standard protrusion simulating bone landmarks and perform an injection from the opposite side of the found point. The participants were asked to use a standardized model for performing IANB (Energiyalab, Moscow, Russia). This model is equipped with a cartridge dental syringe with a needle, a lithium battery, an electrical wiring system, and sensors built into the model. If the technique is performed correctly, a green light signal lights up; if it is incorrect, a red light signal lights up (Figure 2).
4. In the group where the training was provided on the cadavers, the participants determined individual guidelines according to which local anesthesia was performed. Then, the mouth was opened on the cadavers and fixed with a mouth expander. The participants performed intraoral palpation to clarify the injection point and injected from the opposite side in the direction of the finger installed outside the mandible branch. When the inner surface of the mandible branch was reached, an aspiration test was performed an, if not - a local anesthetic solution is injected at a rate of 1 mL per minute.

**Figure 3:**
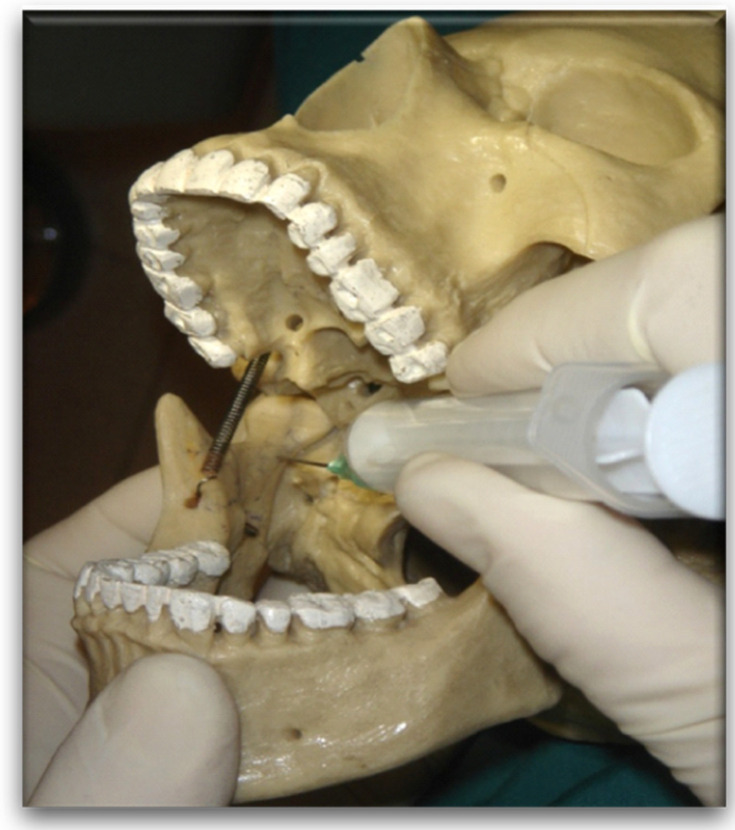
Local anesthesia bone landmarks on the plastic skull.

**Figure 4:**
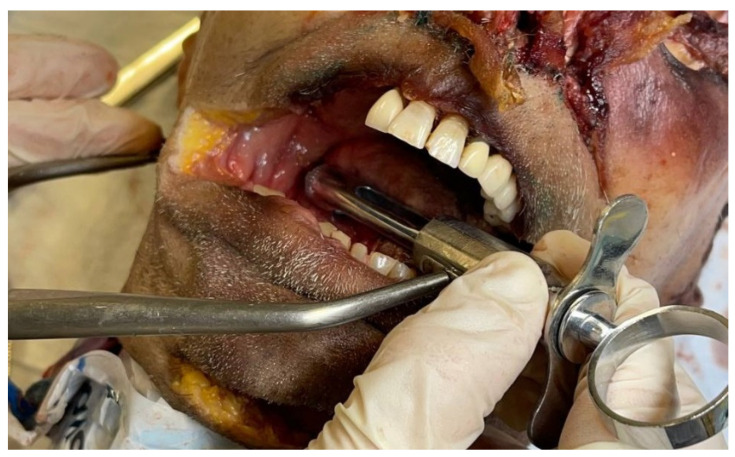
Usage of developing navigation system for IANB performing.

**Figure 5:**
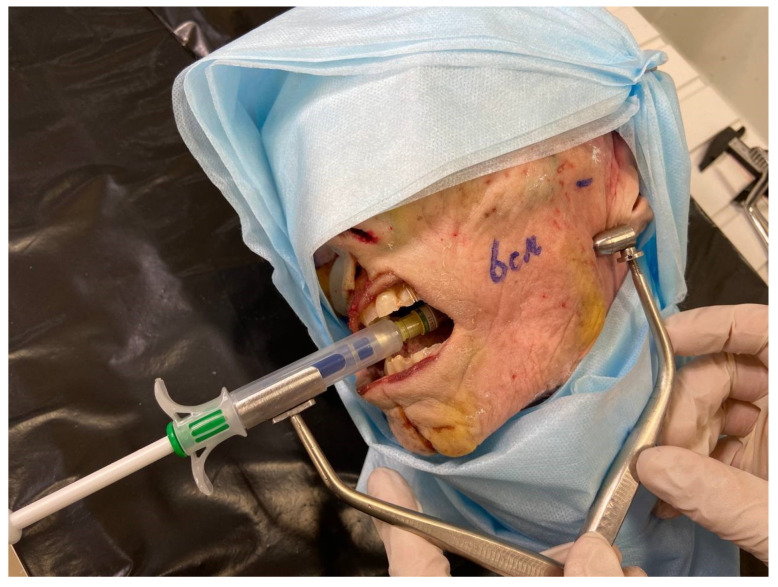
Usage of developing navigation system for IANB, performed by disposable syringe «Artiject».

**Figure 6:**
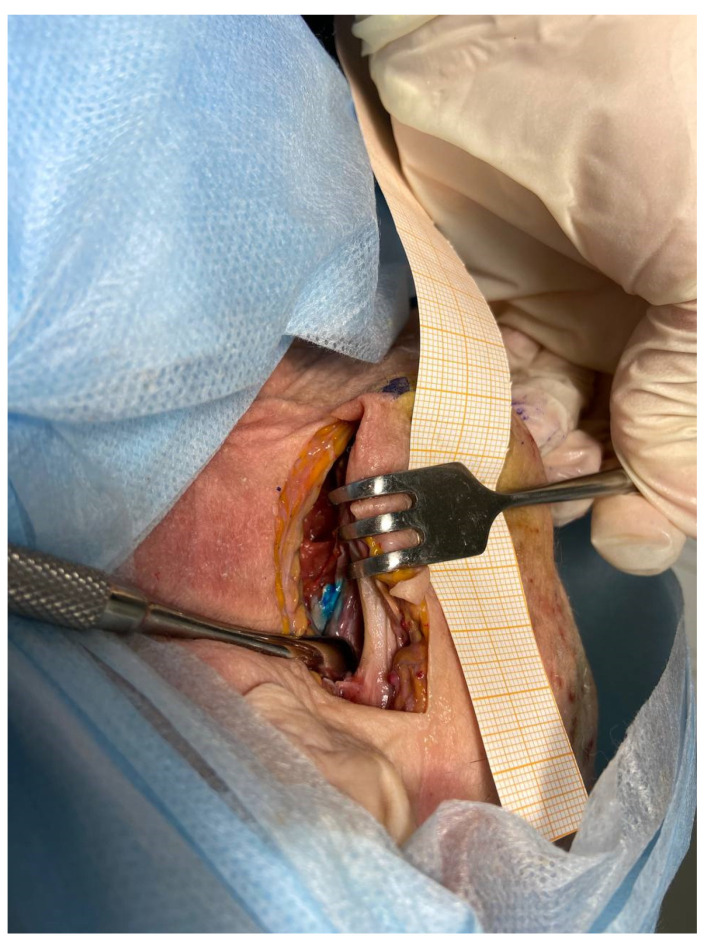
Dissection and injection success verification.

Since each used biological object is an individual workplace, it is impossible to predict the correct injection site in advance. For this purpose, a brightly colored liquid was used as a local anesthetic. This technique made it possible to determine the accuracy of the injection of the solution by dissection after this injection. The use of a biological object was based on 1 head per two participants of the educational program. Anesthesia procedure involved the palpatory determination of bone landmarks, an aspiration test, injection of a local anesthetic solution. For determination of successful injection, the blue silicon introducing and then dissection was performed (Figure 3, Figure 4, Figure 5 and Figure 6).

For the developing navigation device usage, after determining the target point, a syringe was installed in the navigator, and an injection of colored liquid silicone was performed.

At the dissection stage, an incision was made anteriorly from the auricle, and access was made to the space between the lateral and medial pterygoid muscles by layer-by-layer dissection. The presence of colored silicone testified to the success of the injection (Figure 6).

Each type of training was supervised by independent, qualified and certified lecturers, who were not previously familiar with the participants, in order to prevent the impacts of subjective judgment on the results of the study.

An ordinal rating scale (Likert scale) was used to assess satisfaction with aspects of the training conducted in the study [19]. The range of values is from 1 to 5, where 1 is “excellent” and 5 is “awful”. The sum of the scores was also used to analyze overall satisfaction with learning.

Potential bias resource was connected with the objective success of manual skill training in biomaterial assessment, and, also, with the difference in anatomical marks for IANB administration. Although, these biases are more important for possible other research where the rate of successful IANB in dental practice is assessed after these ways of skill training.

Missing data were checked and were not included in the research (twice by 2 researchers, 1% answers)

The results were processed using IBM SPSS Statistics 26 [RUS] and Microsoft Excel 2016 software packages.

The mean, median, standard deviation, and minimum and maximum values were calculated. The normality of the distribution within each sample was evaluated using the Kolmogorov–Smirnov test. With a normal distribution, it was assumed to use parametric statistics methods—Student’s *t*-test, with an abnormal distribution of at least one of the samples—and the Mann–Whitney test. For comparison of the question mark odd ratio (‘excellent’ and ‘good’), the chi-squared test was used, considering the statistical significance as for all results at *p* < 0.05, while α was equal 5%.

## 3. Results

The main group consisted of participants (n = 299), who practiced manual skills on biological material, cadavers (SCIENCE CARE, INC, Phoenix, AZ, USA), from 2017 till 2021 year. The group consisted of 65.6% males and 34.4% females (no gender differences were found in the group of comparison) aged 19 to 73 years (M = 37.66 ± 13.55). The comparison group consisted of participants (n = 700), who practiced manual skills on standardized simulators (plastic models). The group included 13.4% males and 86.6% females aged 20 to 71 years (M = 41.70 ± 12.94).

Due to the significant inequality between the main and the comparison groups, in order to avoid differences using IBM SPSS Statistics 26 [RUS] software, a random selection of 299 observations from the comparison group was carried out to balance the categories of results. The number of participants was counted using the common way and formula, with the results of the other closest research [8,10].

The resulting total research sample (n = 598) for statistical analysis was made up of respondents aged 19 to 73 years (M = 39.45 ± 13.38). The sample included 40.6% males and 59.4% females. The distribution in the resulting sample was of a different character from the normal one (Kolmogorov–Smirnov test, *p* < 0.001), as a result of which the Mann–Whitney U-test was used.

For frequency analysis of participants’ opinions on training (by Likert scale results, ‘1’ is “excellent” and ‘5′ is “awful”), before the final sample (n = 598) was created, scores were used from all participants (n = 299 in the main group and n = 700 in the comparison group).

The obtained results from the main group, which consisted of the questioning of the participants’ (n = 299), according to the group training skills, are presented in Table 1 and Table 2.

Comparison of the excellent-mark rate in groups has shown:

- there was a statistically significant higher rate for satisfaction with participation in the training for cadaver education (9.5 times, *p*-value < 0.001);
- realization of expectations with training for cadaver was seven times more for cadaver education (*p*-value < 0.001);
- training organization satisfaction level was 1.2 times higher for cadaver education (*p*-value > 0.05, with Yates correction);
- education methodology satisfaction was 1.4 times higher for cadaver course (*p*-value < 0.001);
- expectations with education methodology were 2.2 times higher for cadaver course participants (*p*-value < 0.001);
- educational value of education methodology was 1.9 times higher for cadaver training (*p*-value < 0.001);
- manual-skill shaping by education methodology was 3.4 times higher for cadaver IANB training (*p*-value < 0.001);
- tutor satisfaction was 2 times higher for cadaver-training skills than for plastic models (*p*-value < 0.001).

Comparison of the good mark rate in groups has shown the opposite fact; statistically, it was significantly higher in the plastic-model training group for the criteria ‘satisfaction with participation in the training’, ‘realization of expectations with training’, ‘expectations with education methodology’, ‘educational value of education methodology’, ‘manual-skill shaping by education methodology’, and ‘tutor satisfaction’ (*p*-value < 0.001).

Using the Mann–Whitney U-test, significant differences were revealed in such aspects of learning satisfaction as satisfaction from participating in the program, expectations from the program, satisfaction from teaching methods, expectations about teaching methods, educational value of teaching methods, usefulness of teaching methods in terms of mastering knowledge in the future, opinion about the teacher, and overall satisfaction level, in the main and control groups (Mann–Whitney U-test, *p*-value < 0.001). The analysis of the average values allows to say that the respondents from the main group, who practiced manual skills on biomaterial, are more satisfied with the listed aspects of the training and the training in general (Table 3). No differences were found for the opinion on the organization of the program (*p* > 0.05) (Table 3).

## 4. Discussion

The null hypothesis ‘training the IANB manual skill on cadavers is associated with higher total training satisfaction than on plastic models’ was proven as the main part of the assessed criteria of the used scale.

Our null hypothesis is not rejected and was maintained by the fact that cadaver training, regardless of medical specialty, is beneficial [20].

Despite the fact that we did not find analogues of our study in the literature, confirmation of the effectiveness of the chosen trajectory can be found in similar works. We agree that cadavers constitute a high-quality educational tool that, after adequate preparation, allows for practicing an invasive medical procedure [21].

There is no doubt that dissection is the gold standard in anatomy education, but modern pedagogy must combine multiple pedagogical resources [22]. The process of training dentists and the question of choosing the optimal method for providing local-anesthesia manual-skills shaping remain open, despite technological progress and the involvement of innovative technologies.

Despite the fact that the use of cadavers in medical education is an excellent, model, especially in anesthesia [23], according to Chen X. et al., 2018, there are a number of difficulties faced by specialists in the field of dentistry and maxillofacial surgery [24].

In our study, non-fixed cadavers were used, the storage of which was carried out by cooling. The obtained results of the effectiveness of the teaching methodology are confirmed by Lone M. et al., 2017, that cadaveric material, including fixed, thiel-embalmed cadavers, may provide an ideal tool for teaching the injection technique of local anesthesia [25]. Moreover, per Kennel L. et al., 2018, a group of students felt more confident about recognizing anatomy in the living individual, found it easier to identify and dissect anatomical structures, and indicated more active exploration of functional anatomy due to the retained flexibility of the cadaver [26].

The analysis of the average values allows us to say that participants from the main group are more satisfied with the listed aspects and the training in general. No differences were found for the opinion on the organization of the program. In a study conducted by Wong, G. et al. (2020), the authors evaluated the experience gained in manual-skills shaping, including mandibular anesthesia, in dental students based on the results of a questionnaire after applying two techniques—“student-to-student” and “student-to-phantom”. The authors assessed the stress level according to the Interval Scale of Anxiety Response, as well as the level of confidence at the first injection to an adult patient or a child already at a dental appointment, according to the proposed questionnaire. There was no statistical significance in the level of stress and confidence among students who studied, according to the two methods mentioned above. The authors noted that the majority of students preferred the manual training of the skill on their classmates or phantoms [11]. In our study, similar data were obtained.

Collaço E. and co-authors (2021) are engaged in the development and implementation of VR (virtual reality) technologies in the training of dentists, to completely replace manual skills with virtual ones. The authors conducted a study to evaluate the results of teaching mandibular anesthesia to dental students on a virtual-reality device, VIDA Odonto R. In total, 163 students were trained, divided into four groups (depending on the teaching methods): immersive, non-immersive, and their combinations. In addition to analyzing the difference in such physical indicators as the time spent on anesthesia itself, students were surveyed to assess the weaknesses of simulators, the level of confidence, and the accuracy during injections. Participants noted feeling that it was like reality, when working with a virtual syringe, which did not affect clinical practice [13].

Moussa R. (2022), points to such limitations of education “teacher–student–student” and “teacher–student–phantom” as an aspect of the unfinished COVID-19 epidemic that leads to limited contact and increased stress by students, which does not directly depend on the learning process [12,27].

It should be noted that dissection and training using cadaveric material remains the gold standard in medicine. According to Feig G. (2022), autopsies support and improve the necessary knowledge for surgical modules [28]. Cadaveric dissection brings about the skills, courage, and the ability to confidently work on the human body without any fear, for future practice. It is, therefore, recommended that more time should be allocated to cadaveric dissection [29].

### Limitations of the Study

We understand that training program satisfaction has a complex score. We should mention that in this study we did not search for connection between the Lemyr-Tessier-Fillion PSM-25 test [30] participants’ score and any parameters measured in this study, such asnes about participants’ age, sex, academic status, and previous success experience in IANB for practice.

## 5. Conclusions

Important advantages of educational technology using biological material are the sensations of tissue resistance identical to natural ones, the individuality of each object, and the possibility of the visual study of the technique of anesthesia by dissection of the needle course and the location of the anesthetic depot. However, despite the advantages of using biological material for training dentists, the use of this technique has a number of limitations, taking into account the peculiarities of local legislation and the requirements for the organization of the educational process.

## Figures and Tables

**Table 1:** Analysis of the distribution of main group participants’ opinions on training (n = 299).

Scale	“Excellent”, n (%)	“Good”, n (%)	“Satisfactory”, n (%)	“Satisfactory”, n (%)	“Poor”, n (%)
Satisfaction with participation in the program	164 (54.8%)	117 (39.1%)	18 (6%)	0	0
Expectations with training	192 (64.2%)	92 (30.8%)	15 (5%)	0	0
Training organization satisfaction	137 (45.8%)	147 (49.2%)	15 (5%)	0	0
Education methodology satisfaction	149 (49.8%)	146 (48.8%)	4 (1.3%)	0	0
Expectations with education methodology	254 (74.9%)	28 (9.4%)	17 (5.7%)	0	0
Educational value of education methodology	224 (74.9%)	75 (25.1%)	0	0	0
Manual-skill shaping by education methodology	239 (79.9%)	60 (20.1%)	0	0	0
Tutor satisfaction	253 (84.6%)	46 (15.4%)	0	0	0

The obtained results from comparison groups, consisting of the questioning of the participants’ (n = 299), are presented in Table 1. The analysis of the distribution of comparison group participants opinions on training by Likert scale (n = 299) is presented in Table 2.

**Table 2:** Analysis of the distribution of comparison group participants’ opinions on training (n = 299).

Scale	“Excellent”, n (%)	“Good”, n (%)	“Satisfactory”, n (%)	“Poor”, n (%)	“Awful”, n (%)
Satisfaction with participation in the training	17 (5.7%)	248 (82.9%)	34 (11.4%)	0	0
Expectations with training	27 (9.1%)	259 (86.6%)	13 (4.3%)	0	0
Training organization satisfaction	113 (37.7%)	170 (56.6%)	15 (5.1%)	1 (0.6%)	0
Education methodology satisfaction	107 (35.7%)	150 (50.3%)	42 (14%)	0	0
Expectations with education methodology	118 (39.4%)	154 (51.4%)	15 (4.9%)	12 (4.3%)	0
Educational value of education methodology	117 (39.1%)	169 (56.6%)	13 (4.3%)	0	0
Manual-skill shaping by education methodology	71 (23.7%)	228 (76.3%)	0	0	0
Tutor satisfaction	128 (46%)	161 (54%)	0	0	0

**Table 3:** Assessment of the level of satisfaction with training and its aspects in the two groups.

Questions	Comparison Group M ± SD Median Min–Max	Main Group M ± SD Median Min–Max	Significance of Differences (Mann–Whitney Test), Significance Level
Satisfaction with participation in the program	3.93 ± 0.41 4 3–5	4.49 ± 0.61 4.4 3–5	*p* < 0.001
Expectations with training	4.05 ± 0.36 4 3–5	4.59 ± 0.59 4.4 3–5	*p* < 0.001
Education methodology satisfaction	4.22 ± 0.67 4 3–5	4.48 ± 0.53 4.4 3–5	+ *p* < 0.001
Expectations regarding educational methods	4.26 ± 0.74 4 2–5	4.79± 0.53 4.5 3–5	*p* < 0.001
Training organization satisfaction	4.31 ± 0.59 4 2–5	4.41 ± 0.59 4.4 3–5	*p* > 0.05
Educational value of education methodology	4.35 ± 0.56 4 3–5	4.75 ± 0.43 4.5 4–5	*p* < 0.001
Manual-skill shaping by education methodology	4.24 ± 0.43 4.4 4–5	4.8 ± 0.4 4.5 4–5	*p* < 0.001
Educator satisfaction	4.46 ± 0.5 4.4 4–5	4.85 ± 0.36 4.5 4–5	*p* < 0.001
General satisfaction	33.94 ± 1.87 32 26–38	37.16 ± 2.01 35.5 31–40	*p* < 0.001

## Data Availability

The data presented in this study are available on request from the corresponding author.
